# Long Island College Hospital, Brooklyn, N. Y.

**Published:** 1860

**Authors:** 


### Long Island College Hospital, Brooklyn, N. Y.

We have received the announcement of the above Institution, the first course of Lectures in which, will commence on Thursday, 29th March, 1860, and will continue sixteen weeks, and congratulate ourselves, and those who have sustained the policy of the Institution with which we are connected, upon this *triumph* of common sense, justice and propriety, over selfishness, jealousy and folly. This enterprise is undertaken by a number of the most eminent and persevering medical men of the United States, and we heartily wish it the fullest measure of success. The Faculty is composed of the following gentlemen:

Austin Flint, M. D., Prof. of Practical Medicine and Pathology. Frank H. Hamilton, M. D., Prof. of Surgery. Jas. D. Trask, M. D., Prof. of Obstetrics. R. Ogden Doremus, M. D., Prof. of Chemistry and Toxicology. Jos. C. Hutchinson, M. D., Prof. of Surgical Anatomy and Operative Surgery. Jno. C. Dalton, Jr., M. D., Prof. of Physiology and Microscopic Anatomy. Dewitt C. Enos, M. D., Prof. of General and Descriptive Anatomy. Edwin N. Clapman, M. D., Prof. of Materia Medica and Therapeutics. J. G. Johnson, M. D., Demonstrator of Anatomy.

